# A study of the attenuation stage of a global infectious disease

**DOI:** 10.3389/fpubh.2024.1379481

**Published:** 2024-04-05

**Authors:** Tianyi Sun, Baisuo Jin, Yuehua Wu, Junjun Bao

**Affiliations:** ^1^Department of Statistics and Finance, University of Science and Technology of China, Hefei, China; ^2^Department of Mathematics and Statistics, York University, Toronto, ON, Canada; ^3^Endoscopy Center, The First Affiliated Hospital of Anhui Medical University, Hefei, China

**Keywords:** global infectious diseases, varying coefficient SEIR model, kalman filter, attenuation rate, reporting delay

## Abstract

**Introduction:**

Differences in control measures and response speeds between regions may be responsible for the differences in the number of infections of global infectious diseases. Therefore, this article aims to examine the decay stage of global infectious diseases. We demonstrate our method by considering the first wave of the COVID-19 epidemic in 2020.

**Methods:**

We introduce the concept of the attenuation rate into the varying coefficient SEIR model to measure the effect of different cities on epidemic control, and make inferences through the integrated adjusted Kalman filter algorithm.

**Results:**

We applied the varying coefficient SEIR model to 136 cities in China where the total number of confirmed cases exceeded 20 after the implementation of control measures and analyzed the relationship between the estimated attenuation rate and local factors. Subsequent analysis and inference results show that the attenuation rate is significantly related to the local annual GDP and the longitude and latitude of a city or a region. We also apply the varying coefficient SEIR model to other regions outside China. We find that the fitting curve of the average daily number of new confirmed cases simulated by the variable coefficient SEIR model is consistent with the real data.

**Discussion:**

The results show that the cities with better economic development are able to control the epidemic more effectively to a certain extent. On the other hand, geographical location also affected the effectiveness of regional epidemic control. In addition, through the results of attenuation rate analysis, we conclude that China and South Korea have achieved good results in controlling the epidemic in 2020.

## 1 Introduction

In this paper, we examine the decay stage of global infectious diseases. For demonstration, we consider the first wave of the COVID-19 epidemic. The first case of novel coronavirus pneumonia (also known as COVID-19) was reported in December 2019, and it turned into a serious epidemic in 2020 without an effective vaccine or drug treatment. The epidemic then spread rapidly around the world, infecting millions of people by the end of that year. Due to the highly contagious and mutable nature of the virus, the medical research and patient treatment are still ongoing. For more details on COVID-19, see Pak et al. (1), Nalbandian et al. (2), and Chakraborty and Maity (3), among others.

Right after the epidemic began to spread, governments around the world introduced control policies and implemented measures such as school closures and traffic control to reduce the spread of the virus. At the same time, they urged people to take protective measures such as wearing masks and maintaining social distance. Different measures appear to be effective in slowing down the spread of the disease. It is important to find out which elements play the main roles. On the other hand, it is also important to sum up the experience and find the most effective way to control the epidemic.

The epidemic has been analyzed in sociological and medical contexts (4, 5). There are also many studies discussing the reproduction number and other characteristics of COVID-19 in different countries (4, 6–10). Aiming at considering the dynamic spread of the epidemic (11, 12), statistical models and classic epidemic models such as the SIR model (13) or SEIR model (14) have been used at each stage of the epidemic. In recent years, modified SEIR or SIR models have also been proposed and applied to COVID-19 outbreak analysis in China (15–18) and other regions (19, 20).

One of the key questions in this outbreak is the impact of containment measures on the spread and speed of COVID-19. Our work will focus on the post-government response stage and aim to estimate the epidemic dynamics after the implementation of control measures based on a SEIR model of declining infection rates. We introduce the concept of “attenuation rate,” which represents the rate at which infection rates decay under government control. The goal is to consider the evolution of the epidemic during its attenuation stage and to measure the impact of control on the epidemic in different cities. We then test the reliability of the model by fitting the data with the actual infection curve, and analyze the relationship between the attenuation rate and other regional factors.

The rest of this paper is arranged as follows. In Section 2, we present a variable coefficient SEIR model, and then we provide details of the iterative algorithm used to estimate the model parameters and discuss reporting delays (a common problem in epidemiological data). In Section 3, we present simulation results and apply the varying coefficient SEIR model to the 2020 COVID-19 outbreak in Chinese cities. We then conduct statistical analysis of the attenuation rates of Chinese cities, aiming to explore the factors related to the ability of these cities to control the epidemic. We also apply the varying coefficient SEIR model to other epidemic data. We conclude this paper in Section 4.

Regarding the data used in this paper, we note that the data on the number of daily infections are taken from the National Health Commission of the People's Republic of China (21) and the World Health Organization (22), and the population and other data are taken from China's Bureau of Statistics.

## 2 Materials and methods

### 2.1 A varying coefficient SEIR model

The classic susceptible-exposed-infected-recovered (SEIR) model has been applied to case analysis of a variety of infectious disease outbreaks. Among them, susceptible groups, exposed groups, infected groups and recovered groups transform into each other. However, due to the high contagiousness and huge number of infections, the model seems not to work well when applied to the COVID-19 epidemic. Thus, some improvements are made in our model with reference to the work of Li et al. (15).

The infectious disease cases can be divided into two categories: confirmed cases, which are infection cases that are included in official statistics after infection; and unconfirmed cases, which are infection cases that were not included in official statistics because of the mild symptoms. To model an epidemic, the infection rate is one of the most important parameters. It is the number of people that one patient can infect in one day and represents the speed of transmission. Another variable with a similar meaning is the reproductive number *R*, which represents the total number of people a patient may infect. In our modeling, we have *R* = α*βD* + (1 − α)μ*βD*, where α is the proportion of confirmed cases over all cases, β is the infection rate of a confirmed case in one day, μ is the reduction factor of the infection rate of an unconfirmed case compared to that of a confirmed case, and *D* is the average duration of an infection. When focusing on the early stages of an infectious disease outbreak, most studies using infectious disease models (15–17) assumed that the infection rate of the disease is a fixed value, which is reasonable during the free transmission stage. However, this assumption became problematic once local governments began to respond to the epidemic. It can be expected that after the free spread of infectious diseases in the early stages of the outbreak, the infection rate should continue to decline at a certain rate as the government's control measures advance and the public's awareness of prevention and control increases. Therefore, in studying the outbreak of the COVID-19 epidemic, we divide the spread of the epidemic into two stages: the first stage is the free spread stage, and the second stage is when the government implemented control measures. The infection rate as a function of time kept unchanged in the first stage, which is β_0_, but gradually decreased in the second stage. It would be reasonable and convenient to assume that the infection rate of the epidemic continued to decrease at a fixed rate, which implies that the reproduction of the epidemic also decreased at the same rate. Thus, we set up the model M as follows:


$$\begin{array}{l} \frac{dS_{i}}{dt}=-\frac{\beta_{0}S_{i}I_{i}^{r}\tau^{t}}{N_{i}}-\frac{\mu \beta_{0}S_{i}I_{i}^{\mu }\tau^{t}}{N_{i}} \end{array}$$

(1) $$\begin{array}{l} \frac{dE_{i}}{dt}=\frac{\beta_{0}S_{i}I_{i}^{r}\tau^{t}}{N_{i}}+\frac{\mu \beta_{0}S_{i}I_{i}^{\mu }\tau^{t}}{N_{i}}-\frac{E_{i}}{Z} \end{array}$$

$$\begin{array}{l} \frac{dI_{i}^{r}}{dt}=\alpha \frac{E_{i}}{Z}-\frac{I_{i}^{r}}{D}\\ \frac{dI_{i}^{\mu }}{dt}=\left(1-\alpha \right)\frac{E_{i}}{Z}-\frac{I_{i}^{\mu }}{D} \end{array}$$

where *S*_*i*_, *E*_*i*_, $I_{i}^{r}$, $I_{i}^{\mu }$, and *N*_*i*_ are respectively the susceptible population, exposed population, confirmed infected population, unconfirmed infected population, and total population in City *i*, β_0_ is the basic transmission rate in the free transmission stage, τ is the attenuation rate of the daily infection rate, so the infection rate of a confirmed case at time *t* is $\beta_{0}\tau^{t}$, and *Z* is the average latency period.

The approximate solutions of the above simultaneous nonlinear equations can be obtained using the fourth-order Runge-Kutta method. It is noted that the values on the right-hand sides of these equations in Equation (1) are all determined by random samples from Poisson distributions with appropriate parameters to ensure robustness in the actual calculation.

### 2.2 Methodology

#### 2.2.1 Ensemble adjustment kalman filter (EAKF) algorithm

It is well known that any normal distribution is fully characterized by its mean and variance. Therefore, we conduct research under the Gaussian framework, assuming that all random variables in the model obey a normal distribution, and then we can use the mean and variance to characterize their distributions.

In our study, the parameters required for model inference are different in the free spread stage and the attenuation stage. In the attenuation stage, it is no longer necessary to estimate β_0_ as a parameter, but to estimate the attenuation rate τ additionally. In the following, we only discuss the attenuation stage, and the inference in the free spread stage can be carried out analogously.

Before presenting the algorithm in detail, we briefly introduce the variables used in the model, since the following discussion is only for a single city *i*, we omit the subscript *i* in Equation (1). We denote the unobservable variables there by ***X*** = (*S, E*, *I*^*r*^, *I*^μ^, α, τ, μ, *Z, D*)^⊤^ and the daily confirmed new cases by *O*. Further, we divide *X* into two parts: the state variables $X_{\left[\text{s}\right]}={\left(S,E,I^{r},I^{\mu }\right)}^{⊤}$, and the global parameters $X_{\left[\text{g}\right]}={\left(\alpha ,\tau ,\mu ,Z,D\right)}^{⊤}$, where the subscripts [s] and [g] are the indices of the state variable collection and the global parameter collection respectively.

Given the availability of daily number of new confirmed cases, we employ the Ensemble Adjusted Kalman Filter algorithm (EAKF) (23, 24) to obtain the maximum likelihood estimate of the parameters of the Equation (1). First, we introduce the initial setting of the model. Before the first iteration of the algorithm, we set an initial range for each element of ***X***, define the element-wise upper bounds as ***x***_max_ and the element-wise lower bounds as ***x***_min_ which are chosen based on the actual data (An example will be given in Section 3.1). Let Σ be a diagonal matrix such that its *i*th diagonal element is equal to ${\left(\text{the }i\text{th element of }x_{\max,\left[\text{g}\right]}-\text{the }i\text{th element of }x_{\min,\left[\text{g}\right]}\right)}^{2}/4$, *i* = 1, …, 6. In the first iteration, for ℓ = 1, …, *L*, we generate the initial ensemble state member $x_{0}^{\left(\ell \right)}\left[i\right]$ from the uniform distribution *U*(***x***_min_[*i*], ***x***_max_[*i*]), *i* = 1, …, 10. In the *m*th iteration with *m* > 1, we generate the initial ensemble state member $x_{0,\left[\text{s}\right]}^{\left(\ell \right)}$ in the same way as $x_{0}^{\left(\ell \right)}$ in the first iteration, but we generate $x_{0,\left[\text{g}\right]}^{\left(\ell \right)}$ from the multivariate normal distribution with mean vector ${\bar{x}}_{\left[\text{g}\right]}^{m-1}$ and covariance matrix *r*^2(*m*−1)^Σ, ℓ = 1, …, *L*, where ${\bar{x}}_{\left[\text{g}\right]}^{\left(m-1\right)}$ is the sample mean of $\left\{x_{t,\left[\text{g}\right]}^{\left(\ell \right)}\right\}$ in the (*m* − 1)th iteration (see Algorithm 1 for details), and 0 < *r* < 1 is the variance shrinking rate.

**Algorithm 1:** Ensemble adjustment kalman filter (EAKF) algorithm.

**Input:** The number of ensemble units *L*, the number of iterations *M*, the daily number of new confirmed cases at time *t* *o*_*t*,obs_ {*t* = 1, …, *T*}, the variance shrinking rate *r*, the initial range of unobserved variables ***x***_min_ and ***x***_max_, the SEIR framework M (see Equation 1).
**Output:** The estimated global parameters ${\bar{X}}_{\left[\text{g}\right]}^{M}$.
1: $\Sigma =\text{diag}\left[{\left(x_{\max,\left[\text{g}\right]}\left[i\right]-x_{\min,\left[\text{g}\right]}\left[i\right]\right)}^{2}/4\right]$
2: **for** *m* in 1:*M* **do**
3: **for** ℓ in 1:*L* **do**
4: **if** *m* = 1 **then**
5: ${\text{X}}_{0}^{\left(\ell \right)}\left[i\right]\sim U\left({\text{x}}_{\min}\left[i\right],{\text{x}}_{\max}\left[i\right]\right)$, *i* = 1, …, 10 {the dimension of **X** is 10.}
6: **else**
7: $X_{0,\left[\text{s}\right]}^{\left(\ell \right)}\sim U\left(x_{\min,\left[\text{s}\right]},x_{\max,\left[\text{s}\right]}\right)$, $X_{0,\left[\text{g}\right]}^{\left(\ell \right)}\sim N\left({\bar{X}}_{\left[\text{g}\right]}^{m-1},r^{2\left(m-1\right)}\Sigma \right)$
8: **end if**
9: **end for**
10: **for** *t* in 1:*T* **do**
11: $\sigma_{t,\text{obs}}^{2}=\max\left(4,\overset{2}{\underset{t,\text{obs}}{o}}/4\right)$
12: **for** ℓ in 1:*L* **do**
13: $X_{t,\text{prior}}^{\left(\ell \right)}=M\left(X_{t}^{\left(\ell \right)}|X_{t-1}^{\left(\ell \right)}\right)$, $o_{t,\text{prior}}^{\left(\ell \right)}=M\left(o_{t}^{\left(\ell \right)}|X_{t-1}^{\left(\ell \right)}\right)$ {using Equation 1}
14: **end for**
15: ${ō}_{t,\text{prior}}=\frac{\overset{L}{\underset{\ell =1}{\sum }}o_{t,\text{prior}}^{\left(\ell \right)}}{L}$, $\sigma_{t,\text{prior}}^{2}=\frac{\overset{L}{\underset{\ell =1}{\sum }}{\left(o_{t,\text{prior}}^{\left(\ell \right)}-{ō}_{t,\text{prior}}\right)}^{2}}{L-1}$
16: **for** ℓ in 1:*L* **do**
17: $o_{t,\text{post}}^{\left(\ell \right)}=\frac{\sigma_{t,\text{obs}}^{2}}{\sigma_{t,\text{obs}}^{2}+\sigma_{t,\text{prior}}^{2}}{ō}_{t,\text{prior}}+\frac{\sigma_{t,\text{prior}}^{2}}{\sigma_{t,\text{obs}}^{2}+\sigma_{t,\text{prior}}^{2}}o_{t,\text{obs}}+\sqrt{\frac{\sigma_{t,\text{obs}}^{2}}{\sigma_{t,\text{obs}}^{2}+\sigma_{t,\text{prior}}^{2}}}\left(o_{t,\text{prior}}^{\left(\ell \right)}-{ō}_{t,\text{prior}}\right)$
18: **for** *i* in 1:10 **do**
19: $w_{t,\text{prior}}^{\left(\ell \right)}={\text{X}}_{t,\text{prior}}^{\left(\ell \right)}\left[i\right]$, ${\bar{w}}_{t,\text{prior}}=\frac{\overset{L}{\underset{\ell =1}{\sum }}w_{t,\text{prior}}^{\left(\ell \right)}}{L}$
20: $\varsigma =\frac{\overset{L}{\underset{\ell =1}{\sum }}\left(o_{t,\text{prior}}^{\left(\ell \right)}-{ō}_{t,\text{prior}}\right)\left(w_{t,\text{prior}}^{\left(\ell \right)}-{\bar{w}}_{t,\text{prior}}\right)}{L-1}$
21: $w_{t,\text{post}}^{\left(\ell \right)}=w_{t,\text{prior}}^{\left(\ell \right)}+\frac{\varsigma }{\sigma_{t,\text{prior}}^{2}}\left(o_{t,\text{post}}^{\left(\ell \right)}-o_{t,\text{prior}}^{\left(\ell \right)}\right)$
22: ${\text{X}}_{t,\text{post}}^{\left(\ell \right)}\left[i\right]=w_{t,\text{post}}^{\left(\ell \right)}$
23: **end for**
24: ${\text{X}}_{t}^{\left(\ell \right)}={\text{X}}_{t,\text{post}}^{\left(\ell \right)}$
25: **end for**
26: **end for**
27: ${\bar{X}}_{\left[\text{g}\right]}^{m}=\underset{t}{\sum }\underset{\ell }{\sum }X_{t,\left[\text{g}\right]}^{\left(\ell \right)}/LT$
28: **end for**

In light of Li et al. (15), at any time *t* in an iteration, we let the prior distribution of *O* be $N\left({ō}_{t,\text{prior}},\sigma_{t,\text{prior}}^{2}\right)$, where ${ō}_{t,\text{prior}}=\frac{\overset{L}{\underset{\ell =1}{\sum }}o_{t,\text{prior}}^{\left(\ell \right)}}{L}$, $\sigma_{t,\text{prior}}^{2}=\frac{\overset{L}{\underset{\ell =1}{\sum }}{\left(o_{t,\text{prior}}^{\left(\ell \right)}-{ō}_{t,\text{prior}}\right)}^{2}}{L-1}$, and $o_{t,\text{prior}}^{\left(\ell \right)}$ is the output of the number of daily confirmed new cases *O* at time *t* obtained from the SEIR model with the input parameters $X_{t-1}^{\left(\ell \right)}$. We denote the daily number of new confirmed cases at time *t* by *o*_*t*,obs_. We set $\sigma_{t,\text{obs}}^{2}=\max\left(4,\overset{2}{\underset{t,\text{obs}}{o}}/4\right)$ to be the variance of the observation. Then, we are able to update the ℓth ensemble member with the formula of Gaussian convolution as Equation (2) follows:


$$\begin{array}{l} o_{t,\text{post}}^{\left(\ell \right)}\;=\;\frac{\sigma_{t,\text{obs}}^{2}}{\sigma_{t,\text{obs}}^{2}+\sigma_{t,\text{prior}}^{2}}{ō}_{t,\text{prior}}+\frac{\sigma_{t,\text{prior}}^{2}}{\sigma_{t,\text{obs}}^{2}+\sigma_{t,\text{prior}}^{2}}o_{t,\text{obs}} \end{array}$$

(2) $$\begin{array}{l} +\;\sqrt{\frac{\sigma_{t,\text{obs}}^{2}}{\sigma_{t,\text{obs}}^{2}+\sigma_{t,\text{prior}}^{2}}}\left(o_{t,\text{prior}}^{\left(\ell \right)}-{ō}_{t,\text{prior}}\right). \end{array}$$

Since **X** is unknown, for any parameter *w* in **X**, we use its relationship with the daily number of new confirmed cases *O* to update its value as Equation (3) follows:


(3) $$\begin{array}{l} w_{t,\text{post}}^{\left(\ell \right)}=w_{t,\text{prior}}^{\left(\ell \right)}+\frac{\varsigma }{\sigma_{t,\text{prior}}^{2}}\left(o_{t,\text{post}}^{\left(\ell \right)}-o_{t,\text{prior}}^{\left(\ell \right)}\right), \end{array}$$

where $w_{t,\text{prior}}^{\left(\ell \right)}$ and $w_{t,\text{post}}^{\left(\ell \right)}$ are the values of the *i*th ensemble member at time *t* before and after the update, and ς is the sample covariance of {*w*_*t*,prior_} and the {*o*_*t*,prior_}.

The details of the above calculations are summarized in Algorithm 1 below. Since we need to preset a value as the variance of the observation, following the work of Pei et al. (23) and Li et al. (15), we set the variance $\sigma_{t,\text{obs}}^{2}$ to a quarter of the square of the daily number of new confirmed cases *o*_*t*,obs_. To prevent it from being too small, we give a lower bound of 4 in this paper. When we apply our method to real data, in the initialization phase of the model, we set the initial daily number of confirmed cases *o*_0,obs_ to the total number of confirmed cases in the week before and after the first day considered (Considering the effect of the reporting delay). Referring to the work of Li et al. (15), the number of iterations *M* is taken as 10, the number of ensemble state members *L* is set to 300, and the variance shrinking rate *r* is set to 0.9.

#### 2.2.2 A reporting delay

We have assumed that all daily numbers of new confirmed cases are included in official statistics immediately after disease onset in the subsection above. However, due to the limitations of real conditions, statistical data cannot be obtained immediately. There is often a time lag between the occurrence of a case and its inclusion in statistics. Therefore, when conducting simulation experiments and mimicking the real situation, we incorporate the reporting delay *t*_*d*_ into the iterative process to account for this difference. That is, new cases every day need to be moved back *t*_*d*_ days to be included in the iteration.

Reporting delay is defined as the duration from the occurrence of a confirmed case to the date of inclusion in the statistics. It is not a fixed variable, but a random variable. Following Li et al. (15), we assume that the reporting delay is gamma-distributed, which is entirely determined by its shape and mean parameters. By fitting the model with different values of these two parameters, the shape and mean parameters can be selected in real problems. In our example, we find that setting the shape and rate parameters to 1.85 and 0.23 is reasonable, which will result in an expected report delay of about 8.

## 3 Results

### 3.1 Simulation

To evaluate the performance of the proposed model in epidemic modeling, we examine the accuracy of model parameter estimates by setting up synthetic epidemics with different parameter values. We simulate two sets of bursts with different parameter values, one with larger parameter values τ = 0.8, α = 0.5, μ = 0.6, *Z* = 3.0, *D* = 3.0, denoted by Ξ_1_, and the other with smaller parameter values τ = 0.6, α = 0.3, μ = 0.5, *Z* = 2.5, *D* = 2.5, denoted by Ξ_2_. The initial number of confirmed cases is generated based on a discrete uniform distribution between 0 and 1000.

As mentioned in the previous section, before the first iteration of the algorithm, we need to select an initial range for each parameter. In light of Li et al. (15), we let the upper and lower bounds of *S* be *S*_max_ = *S*_min_ = *s*, and the lower bounds of *E*, *I*^*r*^, and *I*^μ^ is $E_{\min}=I_{\min}^{r}=I_{\min}^{\mu }=0$, where *s* is the total population of a city. However, since our inferences involve the decay phase of the epidemic, it is no longer appropriate to set upper bounds on *E*, *I*^*r*^, *I*^μ^ to fixed values as in (15). In order to be closer to the actual situation, based on the approximate ratios of *E*, *I*^*r*^, and *I*^μ^ given in Li et al. (15), we let $E_{\max}=4\times o_{0,\text{obs}},\;I_{\max}^{r}=o_{0,\text{obs}}$, and $I_{\max}^{\mu }=3\times o_{0,\text{obs}}$ using the most recent number of new daily confirmed cases *o*_0,obs_. By taking into account the impact of reporting delays, the initial number of confirmed cases *o*_0,obs_ is set to the total number of confirmed cases in the two weeks closest to the first day of inference.

We now consider the global parameters. In our simulation studies, we find that the estimates of global parameters are sensitive to the initial ranges given beforehand. If we adopt the method in Li et al. (15), we need to set a relatively large initial range for each global parameter, which would result in that if Ξ_1_ is the true set of global parameters, ***X***_[g]_ can be well estimated, and if Ξ_2_ is the true set of global parameters, only the estimates of α and *D* have small biases. After appropriately narrowing the initial range of each global parameter, we obtain an estimate of ***X***_[g]_ with acceptable biases when its true value is Ξ_1_ or Ξ_2_.

**Figure 1:**
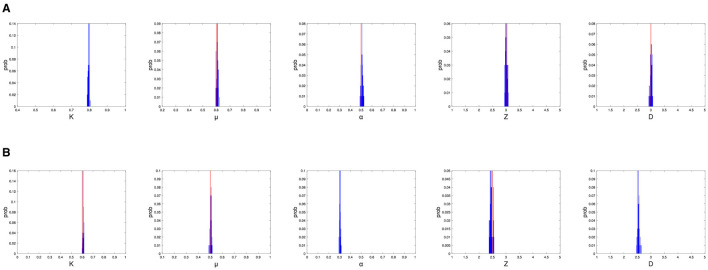
The simulation results, where the global parameter settings are Ξ_1_ (top panel) and Ξ_2_ (bottom panel), and actual parameter values are shown as red lines and distributions of parameter estimates are shown as blue bars. **(A)** The simulation results for Ξ_1_. **(B)** The simulation results for Ξ_2_.

The simulation results, shown in Table 1 and Figure 1, demonstrate that in the model we build, the algorithm can estimate the parameters well given an appropriate initial range. This fact allows us to apply it to real data in the framework of the model.

**Table 1:** The simulation results and the root mean squared errors (RMSE).

**Parameter setting**	Ξ_**1**_			Ξ_**2**_		
	**Actual value**	**Estimation**	**RMSE**	**Actual value**	**Estimation**	**RMSE**
Attenuation rate τ	0.8000	0.7953	0.0058	0.6000	0.6088	0.0098
Reporting rate α	0.5000	0.5077	0.0116	0.3000	0.3045	0.0065
Reduction ratio μ	0.6000	0.6065	0.0090	0.5000	0.5039	0.0070
Infectious period *D*	3.0000	3.0071	0.0341	2.5000	2.5421	0.0542
Latency period *Z*	3.0000	3.0099	0.0369	2.5000	2.4534	0.0669

### 3.2 Real data application

In this subsection, we apply the methodology in Section 2 to the 2020 outbreak in Chinese cities. Because many cities had too few infections then, which would lead to incorrect results, we only consider 136 cities with at least 20 cases there.

Since the Chinese government issued a national-level response on January 23, 2020, and implemented direct measures such as the lockdown of Wuhan, this date divides the 2020 epidemic into two time periods: the period before January 23, 2020, when the epidemic was in the stage of free transmission, and the period after January 23, 2020, the attenuation stage, where the spread of the epidemic was gradually restricted. Our analysis is mainly on the second stage, because there are already many studies on the first stage.

In the attenuation stage, the base value of the transmission rate is 1.12, which is the infection rate we have estimated in the first stage using the SEIR framework [consistent with Li et al. (15)], and the actual infection rate decreased at a fixed rate of τ over time, which, as explained in Section 2.1, represents the percentage reduction in the transmission rate per day. It is obvious that the smaller the τ, the faster the transfer rate will drop. Table 2 displays the global parameter estimates for fifteen cities.

**Table 2:** The global parameter estimates by the varying coefficient SEIR model for selected cities.

**City**	** $\hat{\tau }$ **	** $\hat{\mu }$ **	**Ẑ**	** $\hat{\alpha }$ **	** $\hat{D}$ **
Beijing	0.719	0.505	3.346	0.472	3.265
Shanghai	0.684	0.512	3.054	0.482	3.232
Chongqing	0.717	0.488	3.360	0.445	3.277
Chengdu	0.682	0.518	3.001	0.445	3.259
Suzhou	0.726	0.521	3.451	0.471	3.287
Wenzhou	0.690	0.520	3.231	0.472	3.214
Tianjing	0.782	0.518	3.605	0.495	3.485
Haerbin	0.884	0.581	3.713	0.373	3.575
Ningbo	0.753	0.566	3.656	0.384	3.426
Hefei	0.808	0.528	3.662	0.409	3.516
Fuzhou	0.676	0.523	2.952	0.461	3.097
Zhengzhou	0.764	0.547	3.531	0.463	3.329
Guangzhou	0.758	0.550	3.755	0.365	3.519
textShenzhen	0.727	0.532	3.459	0.421	3.293
Xi'an	0.794	0.551	3.658	0.449	3.482

By Table 2, it can be seen that the infectious attenuation rates of different cities are mostly in the range of [0.7, 0.8], which means that their reproductive numbers *R*_*e*_s drop below 1 after four days and falls to a low level (0.4) a week later. We believe this fact shows that the epidemic has basically been brought under control.

**Figure 2:**
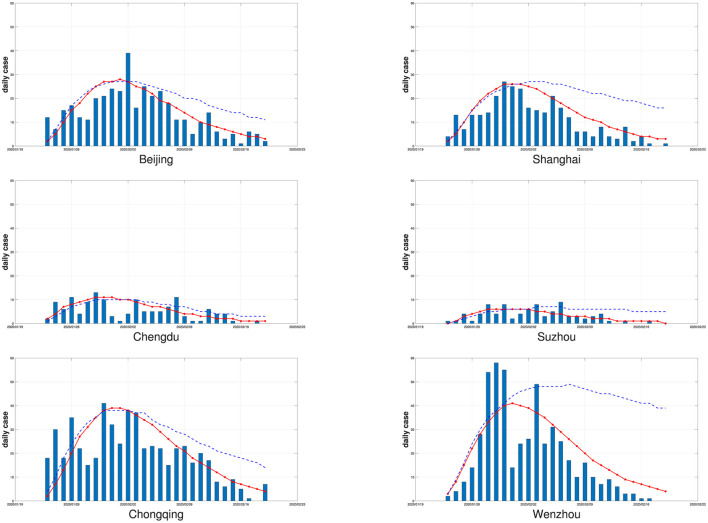
The six cities' daily numbers of new confirmed cases (bars) and the fitted curves of the average simulated daily numbers of new confirmed cases by the varying coefficient SEIR model (solid red line) and the modified SEIR model in Li et al. (15) (dotted blue line) with 100 replicates.

After obtaining the global parameter estimates, we generate the daily number of new confirmed cases by putting the global parameter estimates into our SEIR framework, where the state variables are generated as before. We repeat it 100 times and compute the average simulated daily number of new confirmed cases. Figure 2 displays the actual daily number of new confirmed cases and the fitted curve of the average simulated daily number of new confirmed cases. For comparison with our results, we also apply the modified SEIR model in Li et al. (15) to the epidemic in the corresponding city, and compute the average simulated daily number of new confirmed cases in 100 replicates. The results are shown in the Figure 2, from which it can be seen that the average simulated daily number of new confirmed cases of the modified SEIR model in Li et al. (15) is only in good agreement with the actual number of cases at the early stage of the outbreak, while the average simulated daily number of new confirmed cases from our varying coefficient SEIR model closely matches the number of daily new confirmed COVID-19 cases observed each day throughout the outbreak.

**Figure 3:**
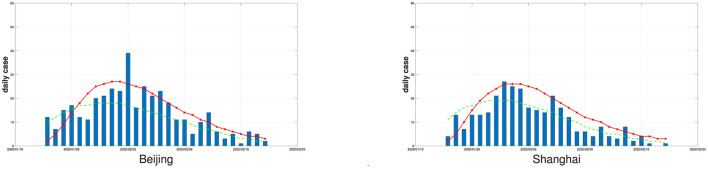
The daily numbers of new confirmed cases (bars) in Beijing and Shanghai and the fitted curves of the average simulated daily numbers of new confirmed cases by the varying coefficient SEIR model (solid red line if the reporting delay is considered, dotted green line if the reporting delay is not considered), the number of replications is 100.

To show the impact of reporting delays, in Figure 3, we display fitted curves of the average simulated daily number of new confirmed cases by the varying coefficient SEIR model with and without considering reporting delays for Beijing and Shanghai. It can be seen that if the reporting delay is considered, the fitted curves are closer to the real data for both cities. Ignoring reporting delays will cause the estimated number of new confirmed cases to peak earlier than the peak of the true number of new confirmed cases, resulting in a non-negligible estimation bias.

### 3.3 Factors associated with the attenuation rate τ

After obtaining the estimate of τ, a common interest is to find out the factors associated with it. Generally speaking, people believe that cities with better economic development conditions are better able to control the epidemic. Strong local governments can effectively improve the efficiency of epidemic control. On the other hand, the attenuation rate may be affected by the location of the city, and some other factors. For demonstration purposes, we use the real data example from the previous subsection.

We consider the following factors: the total local GDP in 2019 and the number of permanent urban residents, which represent the impact of urban development on the attenuation rate; the GDP growth rate in 2019, which represents the capacity of local governments. In addition, we also measure the impact of geographical location by the city's latitude and longitude and the city's distance from Wuhan. Because the size of a local outbreak may affect the difficulty of controlling the outbreak, we include the total number of local cases as a factor.

Since the effect of the attenuation rate τ on the infection rate and the number of daily new confirmed cases is obviously nonlinear, it does not seem appropriate to assume that the attenuation rate is linear with local factors. To find out which local factors have non-negligible correlation with τ, we perform the Spearman correlation test. Considering that the attenuation rate τ is positive, we use logτ in the Spearman correlation test. The Spearman correlation test results between each local factor and logτ based on 136 cities are shown in Table 3, in which the local GDP in 2019, urban latitude and longitude, and urban population in 2019 were all respectively correlated with logτ, except for the significance level of urban longitude, which is <0.1, the significance levels are all <0.05. The remaining issue is deciding whether to include urban longitude as a local factor correlated with logτ. Note that the normality assumption for both logτ and urban longitude is acceptable at the 0.01 significance level after removing a small number of outliers (<5) by Shapiro-Wilk's test. Hence, we test the Pearson correlation between logτ and urban longitude and find that the Person correlation coefficient is 0.1998 and the *p*-value is 0.0197, indicating a non-negligible correlation between logτ and urban longitude. Therefore, we have included urban longitude in the analysis below as well.

**Table 3:** The results Spearman correlation test for each local factor with logτ using data from the 136 Chinese cities.

	**Local GDP**	**Daily No. of new confirmed cases**	**Urban population**	**GDP growth**	**Latitude**	**Longitude**	**Distance to Wuhan**
Correlation coefficient	–0.2395	–0.1377	–0.2272	–0.0204	0.2815	0.1496	–0.1269
*p*-value	0.0051	0.1098	0.0079	0.8141	0.0009	0.0822	0.1410

It can be seen from our analysis that in those 136 Chinese cities, the attenuation rate has a tendency to increase as the regional longitude or latitude increases, which may be affected by the regional climate due to the corresponding changes in longitude and latitude. Furthermore, cities with better economic development tend to exhibit lower attenuation rates, consistent with general thinking that developed economy often indicates strong government execution and perfect public health system. More populous cities have lower attenuation rates, which, contrary to popular belief, may be due to the high correlation between urban population and the local GDP. The Spearman correlation analysis shows that the correlation between the urban population and the local GDP is as high as 0.79, indicating that the correlation between the urban population and the epidemic attenuation rate is affected by the local GDP, and hence urban population can be ignored when modeling the relationship between attenuation rates and other factors. Therefore, we fit a log-linear model of the attenuation rate based only on longitude, latitude, and local GDP. The results are shown in Table 4.

**Table 4:** The log-linear model fit of logτ on the local GDP, and latitude, longitude of 136 Chinese cities.

	**Regression Coefficient**	**Confidence interval**		***T*-test *p*-value**
Local GDP of 2019(trillion yuan)	–0.0347	–0.0506	–0.0188	<0.01
Latitude (degree)	0.0028	0.0010	0.0047	<0.01
Longitude (degree)	0.0023	0.0004	0.0042	0.0188

From Table 4, it is obvious that the local GDP and the longitude and latitude of a city are significant in the log-linear model. The parameter coefficients are –0.0347, 0.00284, and 0.00231, respectively, that is, for every 10 degrees increase in latitude or longitude, the regional attenuation rate increases by 1.0288 times or 1.0234 times. This may be caused by the climate difference between coastal and inland regions in China, similar conclusions can be found in the work of Wu et al. (25) and Srivastava (26). For every 1 trillion yuan increase in the region's total GDP, the attenuation rate would be reduced to 0.9659 times its original value, indicating that the local development level might have an impact on the speed of epidemic containment, which is consistent with Bambra et al. (27).

Although the *p*-value of less than 0.01 for the *F*-test indicates that the model is significant, the fitting performance of our log-linear model is still not good enough, with an *R*^2^ of 0.216, suggesting that the explanatory variables we included in the log-linear model only explain part of the attenuation rate, and the rest may be local subjective factors such as the enthusiasm of the government and residents to control the epidemic. For city *i*, if we set the attenuation rate inferred by the varying coefficient SEIR model to $\tau_{1}^{i}$ and the attenuation rate estimated by the log-linear model to $\tau_{2}^{i}$, then we can evaluate this city's ability to control the epidemic after eliminating objective factors by $\tau_{1}^{i}-\tau_{2}^{i}$. That is to say, for controlling the epidemic, City *i* is considered to have a strong ability to do so if $\tau_{1}^{i}-\tau_{2}^{i}$ is low, otherwise, City *i* is considered to have a poor ability to achieve it.

### 3.4 Application of the varying coefficient SEIR model to other epidemic data

From the real data analysis in Section 3.2 above, we can see that the varying coefficient SEIR model performs well in modeling China's first epidemic in 2020. We are also interested in seeing how the varying coefficient SEIR model performs on other epidemic data. Therefore, in this subsection, we apply the varying coefficient SEIR model to COVID-19 data from other regions in Asia. Subsequently, we also use a log-linear model to fit the attenuation rate. We then compare the attenuation rate estimate from the varying coefficient SEIR model to the prediction from the log-linear model.

An outbreak of COVID-19 infections occurred in Seoul, the capital of South Korea, in early August 2020, and the local government announced on August 16 an elevated quarantine response level to deal with the crisis. Local measures were as harsh as those in China: intercity transport was closed, public gatherings were banned, and schools were required to hold online classes. Using the varying coefficient SEIR model, the attenuation rate of the epidemic in Seoul after August 16 is estimated to be 0.7089, and the log-linear model predicts 0.7038. These two numbers are very close.

**Figure 4:**
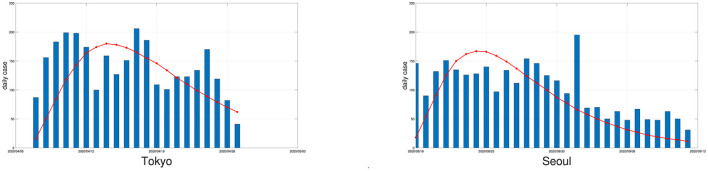
The daily numbers of new confirmed cases (bars) in Tokyo and Seoul and the fitted curves of the average simulated daily numbers of new confirmed cases by the varying coefficient SEIR model (solid red line) with a replication number of 100.

In early April 2020, a large-scale epidemic occurred in Tokyo, the capital of Japan, and the Japanese government declared a state of emergency on April 7, 2020. Based on the daily case data after April 7, 2020, the attenuation rate was 0.6869, compared to the log-linear model's estimate of 0.6309, which means that Tokyo was actually declining more slowly than it should be, most likely because Japan's control measures are much less stringent than those in China and South Korea. Instead of locking down the city, the government only restricted restaurant hours and public events, and urged people to stay indoors. We display the daily numbers of new confirmed cases and the fitted curves of the average daily numbers of new confirmed cases simulated by the varying coefficient SEIR model for outbreaks in Tokyo and Seoul in Figure 4.

India does not release city-specific epidemic data, so we have to use nationwide data to make a rough estimate. The second outbreak in India, which began in April 2021, was a major outbreak in the country. Many local governments announced strengthened prevention and control measures from May 6, 2020. Therefore, we regard this date as the dividing line of the epidemic. By using the approach in Section 2, India's attenuation rate after May 6, 2020, is estimated to be 0.6919. In terms of the GDPs, longitudes and latitudes of several major cities in India, India's attenuation rate would have been around 0.65, which implies that the prevention and control measures implemented in India were not effective enough. This result is consistent with the conclusions of other articles (8, 9) analyzing the effectiveness of control measures in various countries. We believe that during the 2020 epidemic, the control measures taken by China and South Korea were much more effective compared to other countries.

## 4 Discussion

In this paper, we modify the traditional SEIR model by proposing a varying coefficient SEIR model that takes into account daily unconfirmed cases and introduces the attenuation rate. For demonstration, we apply the model to the COVID-19 epidemic in 136 cities in China in 2020 (with more than 20 people infected) and obtain the infection attenuation rates of these cities in the epidemic control stage. We also investigate the relationship between the attenuation rate and other local factors, and find out that the attenuation rate has a significant correlation with the local GDP as well as the latitude and longitude of a city. The results obtained show that the cities with better economic development to a certain extent could control the epidemic more effectively. On the other hand, geographical location also affected the effectiveness of regional epidemic control. In addition to this, we apply the varying coefficient SEIR model to COVID-19 data from other regions in Asia and conclude that China and South Korea achieved better results in controlling the 2020 outbreak.

Our research on the infection attenuation rate can help us more intuitively judge the effectiveness of control measures in different regions, and provide a reference for the effectiveness of control measures when new epidemics occur. However, an important fact derived from the real data example above is that the goodness of fit of log-linear models may not be satisfactory, and the attenuation rate may be affected by other unknown factors, including local government efficiency and public awareness of epidemic prevention and control. At the same time, although the varying coefficient SEIR model can be applied to epidemic data in different countries and periods, due to the differences in reporting delays and other factors in different regions, the use of the attenuation rate estimation of the varying coefficient SEIR model to evaluate the effectiveness of control measures in a country needs to be further studied.

## Data availability statement

Publicly available datasets were analyzed in this study. We collected and collated the original data from the websites: http://www.nhc.gov.cn/xcs/yqtb/list_gzbd.shtml and http://www.who.int/emergencies/diseases/novel-coronavirus-2019/situation-reports. The datasets generated in this study are available upon request from the corresponding author.

## Author contributions

TS: Formal analysis, Methodology, Software, Writing – original draft, Writing – review & editing. BJ: Funding acquisition, Methodology, Writing – original draft, Writing – review & editing. YW: Funding acquisition, Methodology, Writing – original draft, Writing – review & editing. JB: Formal analysis, Methodology, Writing – original draft, Writing – review & editing.

## References

[1] Pak A Adegboye OA Adekunle AI Rahman KM McBryde ES Eisen DP. Economic consequences of the COVID-19 outbreak: the need for epidemic preparedness. Front Public Health. (2020) 8:546036. 10.3389/fpubh.2020.00241 32574307 PMC7273352

[2] Nalbandian A Sehgal K Gupta A Madhavan MV McGroder C Stevens JS. Post-acute COVID-19 syndrome. Nat Med. (2021) 27:601–15. 10.1038/s41591-021-01283-z 33753937 PMC8893149

[3] Chakraborty I Maity P. COVID-19 outbreak: Migration, effects on society, global environment and prevention. Sci Total Environ. (2020) 728:138882. 10.1016/j.scitotenv.2020.138882 32335410 PMC7175860

[4] Tian HY Liu YH Li YD Wu CH Chen B Kraemer MUG. An investigation of transmission control measures during the first 50 days of the COVID-19 epidemic in China. Science. (2020) 368:638. 10.1126/science.abb6105 32234804 PMC7164389

[5] Chen SM Yang JT Yang WZ Wang C Barnighausen T. COVID-19 control in China during mass population movements at New Year. Lancet. (2020) 395:764–6. 10.1016/S0140-6736(20)30421-9 32105609 PMC7159085

[6] Jia JSS Lu X Yuan Y Xu G Jia JM Christakis NA. Population flow drives spatio-temporal distribution of COVID-19 in China. Nature. (2020) 582:389. 10.1038/s41586-020-2284-y 32349120

[7] Kissler SM Tedijanto C Goldstein E Grad YH Lipsitch M. Projecting the transmission dynamics of SARS-CoV-2 through the postpandemic period. Science. (2020) 368:860. 10.1126/science.abb5793 32291278 PMC7164482

[8] Gu J Yan H Huang YX Zhu YR Sun HX Qiu YM. Comparing containment measures among nations by epidemiological effects of COVID-19. Nat Sci Rev. (2020) 7: 5909037. 10.1093/nsr/nwaa243 PMC7543445 34676082

[9] Yan H Zhu Y Gu J Huang Y Sun H Zhang X. Better strategies for containing COVID-19 pandemic: a study of 25 countries via a vSIADR model. Proc R Soc A. (2021) 477:20200440. 10.1098/rspa.2020.0440 35153551 PMC8300607

[10] Leung K Wu JT Liu D Leung GM. First-wave COVID-19 transmissibility and severity in China outside Hubei after control measures, and second-wave scenario planning: a modelling impact assessment. Lancet. (2020) 395:1382–93. 10.1016/S0140-6736(20)30746-7 32277878 PMC7195331

[11] Jones KE Patel NG Levy MA Storeygard A Balk D Gittleman JL. Global trends in emerging infectious diseases. Nature. (2008) 451:990–4. 10.1038/nature06536 18288193 PMC5960580

[12] Morens DM Folkers GK Fauci AS. The challenge of emerging and re-emerging infectious diseases. Nature. (2004) 430:242–9. 10.1038/nature02759 15241422 PMC7094993

[13] Kermack WO McKendrick AG. Contributions to the mathematical theory of epidemics V. Analysis of experimental epidemics of mouse-typhoid; a bacterial disease conferring incomplete immunity. J Hygiene. (1939) 39:271–88. 10.1017/S0022172400011918 20475492 PMC2199446

[14] Hethcote HW. The mathematics of infectious diseases. Siam Rev. (2000) 42:599–653. 10.1137/S0036144500371907

[15] Li RY Pei S Chen B Song YM Zhang T Yang W. Substantial undocumented infection facilitates the rapid dissemination of novel coronavirus (SARS-CoV-2). Science. (2020) 368:489. 10.1126/science.abb3221 32179701 PMC7164387

[16] Sun H Qiu Y Yan H Huang Y Zhu Y Gu J. Tracking reproductivity of COVID-19 epidemic in China with varying coefficient SIR model. J Data Sci. (2021) 18:455–472. 10.6339/JDS.202007_18(3).0010

[17] Wu JT Leung K Leung GM. Nowcasting and forecasting the potential domestic and international spread of the 2019-nCoV outbreak originating in Wuhan, China: a modelling study. Lancet. (2020) 395:689–97. 10.1016/S0140-6736(20)30260-9 32014114 PMC7159271

[18] Prem K Liu Y Russell TW Kucharski AJ Eggo RM Davies N. The effect of control strategies to reduce social mixing on outcomes of the COVID-19 epidemic in Wuhan, China: a modelling study. Lancet Public Health. (2020) 5:E261–70. 10.1016/S2468-2667(20)30073-6 32220655 PMC7158905

[19] Reiner RC Jr Barber RM Collins JK Zheng P Adolph C Albright J. Modeling COVID-19 scenarios for the United States. Nat Med. (2021) 27:94. 10.1038/s41591-020-1132-9 33097835 PMC7806509

[20] Gatto M Bertuzzo E Mari L Miccoli S Carraro L Casagrandi R. Spread and dynamics of the COVID-19 epidemic in Italy: Effects of emergency containment measures. Proc Natl Acad Sci U S A. (2020) 117:10484–91. 10.1073/pnas.2004978117 32327608 PMC7229754

[21] National Health Commission of the People's Republic of China. COVID-19 Data Platform (2020). Available online at: http://www.nhc.gov.cn/xcs/yqtb/list_gzbd.shtml 10.46234/ccdcw2020.082 PMC8392946 34594648

[22] World Health Organization. 2020 WHO Situation Report (2020). Available online at: http://www.who.int/emergencies/diseases/novel-coronavirus-2019/situation-reports

[23] Pei S Kandula S Yang W Shaman J. Forecasting the spatial transmission of influenza in the United States. Proc Natl Acad Sci U S A. (2018) 115:2752–7. 10.1073/pnas.1708856115 29483256 PMC5856508

[24] Anderson JL. An ensemble adjustment Kalman filter for data assimilation. Monthly Weather Rev. (2001) 129:2884–903. 10.1175/1520-0493(2001)129<2884:AEAKFF>2.0.CO;2

[25] Wu Y Jing W Liu J Ma Q Yuan J Wang Y. Effects of temperature and humidity on the daily new cases and new deaths of COVID-19 in 166 countries. Sci Total Environ. (2020) 729:139051. 10.1016/j.scitotenv.2020.139051 32361460 PMC7187824

[26] Srivastava A. COVID-19 and air pollution and meteorology-an intricate relationship: a review. Chemosphere. (2021) 263:128297. 10.1016/j.chemosphere.2020.128297 33297239 PMC7487522

[27] Bambra C Riordan R Ford J Matthews F. The COVID-19 pandemic and health inequalities. J Epidemiol Commun Health. (2020) 74:964–8. 10.1136/jech-2020-214401 PMC7298201 32535550

